# The development and use of Actiphage^®^ to detect viable mycobacteria from bovine tuberculosis and Johne’s disease‐infected animals

**DOI:** 10.1111/1751-7915.13518

**Published:** 2019-12-03

**Authors:** Benjamin M. C. Swift, Nathan Meade, Elsa Sandoval Barron, Malcolm Bennett, Tania Perehenic, Valerie Hughes, Karen Stevenson, Catherine E. D. Rees

**Affiliations:** ^1^ Pathobiology and Population Sciences Royal Veterinary College Hawkshead Herts AL9 7TA UK; ^2^ School of Biosciences University of Nottingham Sutton Bonington Campus Loughborough Leics LE12 5RD UK; ^3^ School of Veterinary and Medicine Science University of Nottingham Sutton Bonington Campus Loughborough Leics LE12 5RD UK; ^4^ Moredun Research Institute Pentlands Science Park Penicuik EH26 0PZ UK

## Abstract

Here, we describe the development of a method that exploits bacteriophage D29 as a lysis agent for efficient DNA extraction from low numbers of mycobacterial cells. This method (Actiphage^®^) used in combination with PCR achieved rapid and sensitive (LOD ≤ 10 cell ml^−1^) detection and identification of viable, pathogenic mycobacteria in blood samples within 6 h. We demonstrate that mycobacteriophage D29 can be used to detect a range of mycobacteria from clinical blood samples including both *Mycobacterium tuberculosis* complex and *Mycobacterium avium* subsp. *paratuberculosi*s without the need for culture and confirms our earlier observations that a low‐level bacteraemia is associated with these infections in cattle. In a study of *M. bovis*‐infected cattle (*n* = 41), the sensitivity of the Actiphage^®^ method was 95 % (95 % CI; 0.84–0.99) and specificity was 100 % (95% CI; 0.92–1). We further used Actiphage^®^ to demonstrate viable *Mycobacterium avium* subsp. *paratuberculosis* is present in the blood of Johne’s infected cattle. This method provides a revolutionary new tool for the study of infections caused by these difficult to grow pathogens.

## Graphical abstract

Actiphage has the ability to rapidly and sensitivity detect pathogenic mycobacteria from the blood of cattle infected with bovine TB and Johne's disease.

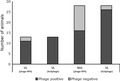

## Introduction

Mycobacteria are responsible for a wide range of diseases in humans and animals. *Mycobacterium tuberculosis* and *Mycobacterium bovis* cause tuberculosis (TB) predominantly in humans and cattle, respectively, although it is known that *M. bovis* can infect a wide range of other animals including humans. *Mycobacterium avium* subsp. *paratuberculosis* (MAP) causes Johne’s disease, a severe wasting disease in ruminants. This disease is endemic in many commercial ruminant herds worldwide (Groenendaal and Zagmutt, 2008) and is recognized as causing significant economic losses to the dairy industry. MAP has also been associated with development of Crohn’s disease in humans (Naser *et al.*, 2014), although a causative link has yet to be conclusively proven.

Bacteria belonging to the genus *Mycobacterium* are typically split into fast‐growing and slow‐growing types, with the fast‐growing bacteria being able to form colonies within 7 days of incubation. In contrast, the slow growers, which typically include the pathogenic species, take more than 7 days to form visible colonies (Wayne and Kubica, 1986; Chacon *et al.*, 2004) and this slow‐growing attribute presents the biggest barrier when studying infections caused by these bacteria. For instance culture of *M. bovis,* MAP and *M. tuberculosis* require incubation times ranging from weeks to months (Plain *et al.*, 2015). Culturing these organisms requires complex media and specialist equipment and is therefore costly in terms of time and reagents. In addition, these bacteria typically exhibit a low plating efficiency, where only a proportion of viable cells in a culture will grow into colonies; thus, culture is not always a reliable way to detect low numbers of cells present in a sample (Messelhausser *et al.*, 2012; Fawzy *et al.*, 2015). This problem is exacerbated by the need to treat clinical samples with harsh chemicals to inactivate contaminating microbes that will overgrow samples during the long incubation periods, but also reduces the viable population of mycobacteria (Grant *et al.*, 2003; Medeiros *et al.*, 2012). Despite these limitations, culture‐based methods are still considered to be the gold standard for diagnostic purposes (Raffo *et al.*, 2017), even though it may not be as sensitive as other diagnostic tests for detecting infection.

These difficulties mean that alternative, more rapid detection methods for mycobacteria are often used. PCR has proven to be a powerful tool for the specific detection of mycobacterial signature DNA sequences from a variety of sample matrices (Dinnes *et al.*, 2007; Priyadarshini *et al.*, 2017), and most recently loop‐mediated isothermal amplification (LAMP) methods have been successfully developed for the specific detection of MAP (Punati *et al*., 2019). However, when used as a diagnostic test, efficient release of DNA is crucial for the success of any PCR or nucleic acid‐based method, especially when this is being used to quantify the number of cells present in a sample. Mycobacteria have mycolic acid‐rich cell walls which are physically robust and difficult to rupture, and this can adversely affect the sensitivity of PCR‐based detection methods (Vandeventer *et al.*, 2011). Inhibitors present in different sample matrices can also affect PCR sensitivity, especially when the sample originates from clinical specimens such as whole blood. Another problem is that standard PCR detects all DNA and therefore does not discriminate between material originating from live or dead cells (Yang and Rothman, 2004), which limits its use to monitor disease clearance or treatment. Advances are being made with electrochemical‐based sensors for human diagnostics, which can be performed independently of culture, but are not currently developed for use in animals (Golichenari *et al.*, 2019). Currently, there is no routine, highly sensitive method that combines the specificity and rapidity of PCR with the live/dead differentiation of culture.

Bacteriophage (phage) are viruses that are natural predators of bacteria, and phage‐based detection methods for mycobacteria were first described for the detection of *M. tuberculosis* in humans (Rees and Botsaris, 2012). One such assay has been termed phage amplification and results in the formation of plaques in a lawn of the fast‐growing non‐pathogen *M. smegmatis* (Stewart *et al.*, 1998)*.* We previously showed that the DNA released from the mycobacterial cells detected at the end of the assay could be extracted from the plaques and used as a template for amplification of mycobacterial signature sequences (phage‐PCR) (Stanley *et al.*, 2007). In further studies, phage‐PCR or phage‐RPA (recombinase polymerase amplification) has been used to detect different mycobacterial pathogens, including members of the *M. tuberculosis* complex group of organisms, as well as MAP, in blood and milk samples from naturally infected animals (Stanley *et al.*, 2007; Botsaris *et al.*, 2010; Swift *et al.*, 2013; Swift *et al.*, 2016a). In these studies, identification and enumeration of slow‐growing mycobacteria was possible within 2 days. However, the method is laborious, requires the overnight incubation of bacterial culture plates and is not suitable for high‐throughput testing. A specific problem is the mechanical handling and transfer of materials into the different types of containers required for the different stages of the assay, which can lead to loss of sample and reduced sensitivity of detection (Swift *et al.*, 2016a).

Phage commonly encode enzymes to efficiently break open cells to release the progeny phage at the end of their replication cycle (Rodriguez‐Rubio *et al.*, 2016). It was hypothesized that a new method could be developed to detect low numbers of mycobacteria by utilizing phage to efficiently lyse the host cells in a liquid sample and then using DNA amplification methods to sensitively detect the DNA released from these cells. This would remove the requirement to amplify the detection event by using lawns of fast‐growing mycobacteria. In addition, this approach would be simpler, faster and should increase sensitivity of the original phage‐PCR method as there is less opportunity for sample loss. Here, we present the development of a rapid detection method (Actiphage^®^) that uses phage to lyse the mycobacteria cells in a liquid system. The sensitivity of the new method compared with phage‐PCR was then determined using clinical blood samples.

## Results

### Determining the eclipse phase of bacteriophage D29

Phage D29 is a well‐studied and characterized mycobacteriophage with a broad‐host range that is capable of infecting wide range of pathogenic and non‐pathogenic mycobacteria (Dedrick *et al.*, 2017). The proposed basis of the new Actiphage^®^ method is that viable mycobacteria release DNA during the lysis event that occurs at the end of the D29 lytic cycle, and this DNA can be detected by PCR to identify the cell type present in the sample. David *et al. *(1980) showed that the growth rate of D29 is related to the growth rate of the host cells; therefore, it was important to determine the time required for D29 to complete its replication period and induce bacterial cell lysis for different hosts under the conditions used for the phage assays. This was achieved using a modification of the phage amplification method to determine the length of the eclipse phase (time between infection and bacterial cell lysis) for different types of mycobacteria. The results (Fig. S1) showed that new phage particles began to be released from infected *M. smegmatis* and *M. bovis* BCG cells after approximately 120 min. The eclipse phase for MAP was longer with phage particles released after 135 min. From this, it was determined that after addition of phage, and allowing for asynchronous infection events, an additional 45 min of incubation (180 min total incubation) would be sufficient to allow D29 to complete its replication cycle and fully release the genomic DNA from all of the different types of mycobacterial cells present in a sample.

### Establishment of the principles of the Actiphage^®^ method

**Figure 1:**
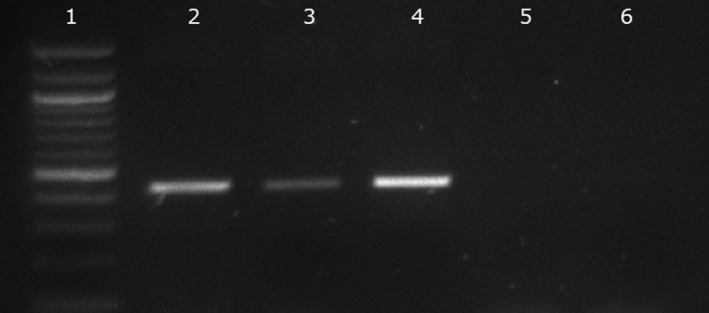
Detection of MAP DNA with and without phage lysis. The effect of centrifugation (Lanes 2 and 3) and filtration (Lanes 4 and 5) on the PCR amplification of signature IS900 sequences of MAP. Lane 1 is the molecular marker (100 bp ladder). In lanes 2 and 4, the MAP cells were lysed using phage, and in lanes 3 and 5, no phage was added to the sample. Lane 6 is the no template control.

The results showed that following lysis with the phage, DNA released from both MAP strains used was detected (Fig. 1). However, a small amount of PCR product was detected in the phage‐negative control indicating that some DNA was being detected due to heat lysis of intact cells during PCR (Fig. 1, lane 3). While this experiment confirmed that the host DNA was preserved sufficiently in a liquid phage lysate to allow later detection by PCR, it suggested that centrifugation was not an effective method to remove any remaining intact cells. To resolve this problem, after incubation with the phage the mixture containing lysed cells and phage (100 µl) was passed through a 0.22‐µm filter to remove any remaining intact cells from the lysate and the DNA cleaned and concentrated as before. Using this separation method, PCR amplification of the signature sequences now only occurred in the samples to which the phage had been added, indicating that the release of DNA was due to phage replication in the viable cells (Fig. 1, lane 4).

### Determining the Limit of Detection (LOD) of the Actiphage^®^ method in blood

To determine whether the Actiphage^®^ method was capable of detecting mycobacteria recovered from PBMCs, cultures were diluted and samples ranging from 10^4^ to 0 pfu ml^‐1^ (rapid enumeration of cell number was performed using the phage amplification assay; 2.3). In this experiment, a recent clinical isolate of *M. bovis* recovered from an infected badger was used to represent a strain that had not been continuously passaged in the laboratory was included in the test set as well as laboratory strains of MAP and *M. bovis* BCG. The diluted samples of bacteria were mixed with whole sheep blood for 4 h to allow internalization of the mycobacteria within PBMCs (Bower *et al.*, 2010), and then, PBMCs were purified using ficoll gradients. The purified PBMCs were resuspended in modified 7H9 media which results in lysis of the PBMCs and release of any intracellular mycobacteria into the media (Swift *et al.*, 2016b; Donnellan *et al.*, 2017). Samples were tested using either the phage amplification or the Actiphage^®^ method. The results (Table 1) showed that the Actiphage^®^ method was able to detect fewer cells than the original phage amplification method for MAP and the clinical isolate of *M. bovis*, with a LOD of < 10 cells. For the *M. bovis* BCG strain, the detection limit was the same for both methods.

**Table 1:** Determining the Limited of detection of the Actiphage assay.

Number of cells^a^	MAP^b^		*M. bovis* BCG^b^		*M. bovis* b	
	Phage assay	Actiphage^®^ method	Phage assay	Actiphage^®^ method	Phage assay	Actiphage^®^ method
10^4^	+	+	+	+	+	+
10^3^	+	+	+	+	+	+
10^2^	+	+	+	+	+	+
10^1^	+	+	+	+	+	+
10^0^	−	+	+	+	−	+
0	−	−	−	−	−	−

Results represent the detection of each mycobacteria in three independent samples. For both the phage amplification assay and the Actiphage^®^ method, ‘+’ denotes positive molecular detection in all three replicates;’−‘ denotes no detection of mycobacteria in all three replicates.

a Number of cells added to each sample was determined using the phage amplification enumeration method described by Rees and Botsaris (2012).

b For details of the PCR assay used for the different types of bacteria see the method section.

### Improved sensitivity for detection of *M. tuberculosis* complex in naturally infected SICCT‐positive animals

**Figure 2:**
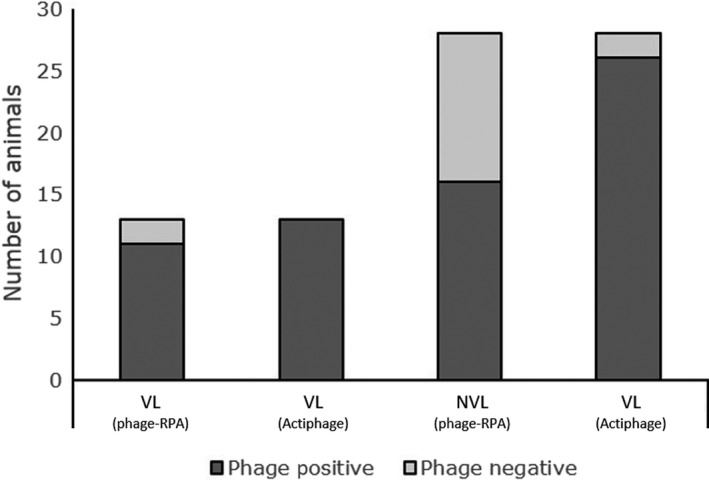
Comparison of ability of phage‐RPA assay and Actiphage® method to detect M. bovis in blood of cattle. Detection of M. bovis in the blood of cattle stratified by SICCT status and lesion status (visible lesions; VL, or non‐visible lesion, NVL). Phage‐positive samples are in dark grey, and phage‐negative samples are in light grey. The results gained using the Actiphage® method are compared with the previously published results using the phage‐RPA assay (Swift *et al.*, 2016b).

In our previous paper, we reported that 66 % of blood samples from Single Intradermal Comparative Cervical Tuberculin (SICCT) test‐positive cattle had detectable levels of MTB complex in their blood using phage‐RPA (Swift *et al.*, 2016a). A second blood sample from these SICCT‐positive cattle was tested in parallel using the Actiphage^®^ method and now DNA from MTB complex bacteria was detected in 95 % (39/41) of the samples (Fig. 2). There was a 100% correlation between the phage negatives, but when assessed overall, these data demonstrate a weak positive correlation between the original phage assay and the Actiphage^®^ assay (*r* = 0.31; 95% CI: 0.01–0.57), where significantly more MTB complex bacteria were detected using Actiphage^®^ (*P* < 0.001). These SICCT‐positive animals had been classified post‐mortem as either Visible (VL) or non‐visible lesion (NVL), and the data showed that the Actiphage^®^ method detected MTB complex bacteria in the blood of all (*n* = 13) of the VL animals and 93 % (26/28) of the NVL animals. In comparison, the original phage‐RPA method detected only 11/13 and 16/28 for the VL and NVL groups respectively. Interestingly, both of the animals that gave a negative result using the Actiphage^®^ method were also negative when tested using the phage‐RPA method. Results for blood samples from a negative control group (45 animals) all gave negative results using both methods, indicating that the increased detection rate achieved using the Actiphage^®^ method was not due to decreased specificity. Using the SICCT status as a comparator, the Actiphage^®^ phage assay delivered a sensitivity of 95 % (95 % CI; 0.84–0.99) and specificity of 100 % (95 % CI; 0.92–1).

### Comparison of Actiphage^®^ with phage‐PCR detection of MAP in cattle blood

We have previously shown that phage‐PCR can be used to detect MAP in the blood of naturally infected cattle (Swift *et al.*, 2013). To confirm that the new Actiphage^®^ method was also able to detect MAP in blood, samples were obtained from experimentally infected cattle that were part of an on‐going study which included 15 animals that had been orally inoculated with MAP F13 and also from eight matched, uninfected negative control animals. The blood samples were taken 9 months post‐infection for analysis by commercial ELISA (IDEXX) and by both the Actiphage^®^ method and the phage‐PCR method. For the phage assays, PBMCs were purified from 2 ml of whole blood which were then split into two parallel samples after they had been resuspended in modified 7H9 media to release any intracellular mycobacteria.

Culture of intestinal tissues was carried out at necropsy to provide evidence that infection was established. MAP was detected in the tissue samples from all but one animal in the infected group (Animal #1, Table 2A), whereas MAP was not cultured from the intestinal tissues of the animals from the control group (Table 2B). Using the phage‐PCR method, low numbers of viable MAP cells were detected in 40 % (6/15; average number of cells detected = 4.5 per 1 ml blood) of animals in infected group and none in the control group. In contrast, the Actiphage^®^ method identified detectable levels of MAP cells in the blood of 87% (13/15) of the experimentally infected calves. Since the majority of animal in this group had a confirmed MAP infection, again this suggests that the Actiphage^®^ method is more sensitive than the original phage‐PCR method. However, using the Actiphage^®^ method, MAP was also detected in two of the blood samples from the negative control group (Table 2, panels A and B). Interestingly, none of the calves in either the infected or control group showed a positive response using a commercial Johne’s disease antibody ELISA test (IDEXX) at necropsy (Table 2).

**Table 2:** Detection of MAP in experimentally infected calves.

Assigned number	Phage–PCR assay				
	MAP culture from tissue	No. of plaques^a^	MAP‐PCR (+/−)^b^	Actiphage^®^‐MAP	ELISA^c^
A					
1	−	1	−	−	−
2	+	1	+	+	−
3	+	0	N/A	+	−
4	+	0	N/A	−	−
5	+	0	N/A	+	−
6	+	0	N/A	+	−
7	+	0	N/A	+	−
8	+	1	+	+	−
9	+	0	N/A	+	−
10	+	0	N/A	+	−
11	+	5	+	+	−
12	+	15	+	+	−
13	+	3	+	+	−
14	+	2	−	+	−
15	+	9	+	+	−
B					
1	−	11	−	−	−
2	−	5	−	−	−
3	−	12	−	+	−
4	−	4	−	−	−
5	−	12	−	+	−
6	−	0	N/A	−	−
7	−	0	N/A	−	−
8	−	8	−	−	−

+, denotes a MAP‐positive test result; −, denotes a MAP‐negative test result.

a In the phage‐PCR assay, the number of plaques formed is indicative of the number of viable mycobacteria detected; detection of specific pathogens by the assay is confirmed by PCR.

b Samples are only considered to be MAP‐positive in the phage‐PCR assay if IS900 is detected by PCR in DNA extracted from plaques. N/A indicates that no plaques were formed; therefore, the result was scored as negative (no mycobacteria detected), and therefore, no PCR was performed.

c IDEXX Johne’s ELISA.

As the Actiphage^®^ method detected MAP from two samples in the negative control group, a question remained about the specificity of the Actiphage^®^ MAP assay. Therefore, further testing was carried out using superfluous DNA samples prepared from the negative control herd used for the study of SICCT‐positive cattle described in section 3.4 as this herd was also known to be free of Johne’s disease, as they had not had a positive Johne’s test animal for at least 5 years. In this case, none of 45 samples tested gave a positive IS900 PCR result.

## Discussion

We have previously described the use of phage amplification‐PCR or phage‐RPA to reduce the time to detection of viable mycobacteria in blood to 48 h (Swift *et al.*, 2013); however, this method requires multiple transfer steps, the incubation of culture plates for 18 h and manual extraction of DNA from agar, making it unsuitable for rapid, high‐throughput analysis. When developing the Actiphage^®^ assay, some of the principles of the original phage‐PCR assay have been retained. For example, previous research has shown that using 10^8^ phage per ml in each sample ensures that very low numbers of mycobacterial cells are productively infected by the phage. This is simply a probability event of guaranteeing two very small particles interact in the (relatively) large sample volume, and at low host cell density, there is no evidence that this results in lysis from without or abortive infection (McNerney *et al.*, 2004). The aim of these experiments was to develop a new, simpler version of the phage‐PCR amplification assay that was capable of sensitively detecting viable mycobacteria in blood samples within 1 day. We first stablished the length of time required for phage D29 to complete its lytic cycle in different host cells and established that this did result in release of chromosomal DNA into the phage lysate that could be used for the detection of signature sequences, despite the extended incubation period and absence of agar that might protect the DNA in the original phage‐PCR assay format. However, we also found that carry‐over of intact cells into PCRs can also result in cells lysis, and release of detectable DNA. Introducing a filtration step ensured that any unlysed cells present in the sample did not contaminate the DNA released from the viable cells so that a positive signal was only achieved when viable cells were present. Using a combination of phage lysis with filtration (termed the Actiphage^®^ method), limit of detection studies shows that D29 can be used as an efficient cell lysis agent that only targets viable cells for a range of different mycobacterial species. The use of phage D29 in a closed system means that there is no driver for phage resistance, which can occur in phage‐therapy treatments when the bacteria are continuously exposed to phage over long periods of time and outgrowth of resistant mutants can occur. The potential limitation exists that the method may not detect phage‐resistant isolates present in blood samples, although resistance to this particular phage has never been reported. This could be mitigated against by using cocktail of phage in these assays. The fact that Actiphage^®^ only detects viable cells ensures that when the bacteria are detected in blood, we can be confident that an animal is currently infected, and we are not just detecting free DNA or the DNA from mycobacteria that may have been killed by macrophage or detecting an immune response still present after infection has been controlled.

The method was then applied to two sets of clinical blood samples, and we could show that it could detect viable pathogenic mycobacteria within 6 h. For the samples from SICCT‐positive cattle, we were able to compare the results gained using the Actiphage^®^ method with those reported earlier using the original phage amplification method. A marked increase in the number of positive samples detected was seen (39/41 from 27/41), with all of the VL animals and the majority of the NVL animals now giving a positive result. Since the specificity of the SICCT is high [99.91 % (±0.013)] for severe interpretation (Goodchild *et al.*, 2015), there is a strong probability that these animals were infected with *M. bovis.* Two samples did not give a positive results using Actiphage^®^. Interestingly, these two samples were also negative in our original study using the original phage‐RPA method and suggest either these animals had not developed bacteraemia or that the number of cells in the blood was below the limit of detection of the assay. The increased number of positive results gained in the group with known infection increased using the Actiphage^®^ method strongly suggests that, as expected, the new method is more sensitive than phage‐PCR because the sample material remains in one tube throughout the test and there is less potential for sample loss. In contrast, none of the samples from the negative control group gave a positive result using either method (*n* = 45), indicating that the increased sensitivity is not due to a reduction in test specificity.

The Actiphage^®^ method was also shown to be able to detect MAP in clinical blood samples from experimentally infected cattle. The results showed that 87 % (13/15) of experimentally infected animals were infected using the Actiphage^®^ method, and again, the method was more sensitive than phage‐PCR. In this study, the Actiphage^®^ did also detect MAP in two of the negative control group and there is no obvious explanation for this result. However, when a further 45 samples from a herd with no history of MAP infection were tested, all these samples gave a negative IS900 PCR result, suggesting that these false‐positive results may have occurred due to a particular circumstance associated with this infection study rather than due to the specificity of the Actiphage^®^ method when detecting MAP.

The power of this assay is that only the presence of mycobacteria is needed for Actiphage^®^ to give a positive result, and therefore, it can be applied to any species of animal as there is no reliance on a host immune response. Recently, the Actiphage^®^ method has been used to detect *M. tuberculosis* in the blood of human patients (Verma *et al.*, 2019). It was also interesting that in the calf infection study described here, no antibody response was detected in any of the animals with clear evidence of systemic infection, although this may reflect a limitation of the ELISA assays to detect Johne’s disease as reported by Nielsen and Toft (2008).

Although there are molecular methods capable of identifying signature DNA from mycobacteria within a day, the sensitivity of these methods can be compromised by the inefficiency of DNA extraction methods (Thakur *et al.*, 2011) and most do not distinguish between live and dead cells, which can result in misinterpretation of treatment efficacy and diagnosis (Alli *et al.*, 2011). Rapid detection of low numbers of viable slow‐growing mycobacteria has generally required amplification of cell numbers by liquid culture‐based methods or immunomagnetic separation to concentrate cells and improve subsequent culture (Stewart *et al.*, 2013), but such methods are still limited by the slow growth of these bacteria. Reverse transcriptase PCR methods exist for some mycobacteria but isolation of RNA from clinical samples is problematic (Alli *et al.*, 2011).

Actiphage^®^ offers the potential for rapid, inexpensive, sensitive detection of live mycobacteria in high‐throughput format that reduces the time to detection of slow‐growing mycobacteria from months to hours. The sensitivity of the method allows detection of low numbers of cells in clinical blood samples and therefore has the potential to revolutionize research into mycobacterial infections.

## Experimental procedures

### Strains, culture and clinical samples

Laboratory strains of mycobacteria used in this study were *M. smegmatis* (mc^2^155); *M. bovis* BCG (cv Pasteur); *M. bovis* (clinical badger isolate) MAP strains K10, ATCC 19698 and B4 (clinical isolate). A low passage UK MAP field strain (F13) was used for the experimental calf infections. Both *M. bovis* (including BCG) and MAP were cultured and maintained on Middlebrook 7H11 and 7H10 agar supplemented with OADC (Becton Dickenson, Swindon, UK), respectively, and liquid cultures were prepared in Middlebrook 7H9 media supplemented with OADC. All cultures were cultured at 37°C without shaking (to prevent clumping). *M. smegmatis* was cultured for up 48 h, *M. bovis* for up to 8 weeks and MAP for up to 16 weeks. Liquid cultures of MAP were supplemented with Mycobactin J (2 µg µl^‐1^; Synbiotics Corporation, Lyon, France). Cultures were confirmed as MAP or *M. bovis* by end‐point PCR (Techne Thermocycler, Staffordshire, UK) targeting IS*900* and IS*6110* specific genetic elements respectively. Bacteriophage D29 (supplied by PBD Biotech Ltd, Suffolk, UK) was used to infect the mycobacteria. When performing phage assays, Modified 7H9 media was used which consisted of Middlebrook 7H9 media supplemented with 2 mM CaCl_2_. All cultures were grown at 37°C without shaking, but with a head space ratio of at least 1:5.

### Isolation of peripheral blood mononuclear cells (PBMCs) from clinical samples

For method optimization commercial sheep blood (Oxoid, UK) was used. Clinical whole blood samples were obtained after slaughter in Vacutainer sodium‐heparin tubes (Becton Dickenson). The isolation of the PBMCs was carried out using Ficoll‐Paque Plus according to the manufacturer’s instructions (GE Healthcare Life Sciences, Amersham, UK). The buffy coat layer was recovered, and the PBMCs were washed in 6 ml of PBS, then recovered by centrifugation (100* g*, 10 min, 18°C). The PBMC cell pellet was resuspended in modified 7H9 media (1 ml). These samples were then centrifuged (13 000 *g*; 3 min), and the bacterial cell pellet was finally resuspended in modified 7H9 media (100 µl) prior to performing the Actiphage^®^ assay.

### Detection and enumeration of mycobacteria and bacteriophage

Phage D29 suspensions were titred on a lawn of *M. smegmatis* grown in soft Middlebrook 7H10 agar (0.75 % w/v) and incubated at 37°C for 18 h. The phage amplification assay and experimental controls were carried out according to Swift *et al.*, 2016a. Briefly, processed blood samples were mixed with 10^8^ D29 bacteriophage to ensure every mycobacteria in the sample were infected and incubated for 1 h to allow the phage to infect any mycobacteria present in the sample. Phage that had not infected their host cells was inactivated using a virucide (ferrous ammonium sulfate; 10 mM final concentration) and the samples were mixed with approximately 10^9^ M*. smegmatis* cells and 5 ml modified 7H9 media before plating using soft 7H10 agar (0.75 % w/v agar final). The number of mycobacterial cells detected in the original sample was determined by counting the number of plaques on the lawn of *M. smegmatis* cells (data reported as pfu ml^‐1^). Enumeration of mycobacterial cells present in laboratory cultures was performed using a modification of the phage amplification assay which included serial dilution of the samples prior to plating, and the number of viable mycobacteria cells detected was reported as pfu ml^‐1^. For detailed schematics, see Rees and Botsaris (2012).

The length of the eclipse phase for D29 was determined using a second modification of the phage amplification. Briefly, approximately 1 x 10^2^ cells of each host strain were mixed with phage D29 (m.o.i. 10) and incubated at 37°C for 30 min. Virucide was added to inactivate any free phage, and after 5‐min contact time, the virucide was diluted by the addition of 5 ml of modified 7H9 media. Samples were removed at different time points and centrifuged (13 000 *g*; 3 min) to remove bacterial cells and the number of free phage particles determine by phage titration on a lawn of *M. smegmatis*.

### Actiphage^®^ detection method

For all cattle tested, PBMCs were isolated from whole blood (Section ‘Determining the Limit of Detection (LOD) of the Actiphage® method in blood’) and were lysed by resuspending in 100 µl modified 7H9 media. Phage D29 (approximately 10^7^ phage particles in 10 µl) was added to the sample, giving the same final pfu ml^‐1^ as using in the original phage amplification assay and incubated for 3 h at 37°C. After incubation, the samples were filtered at room temperature using a microcentrifuge tube filter (0.22 µm pore size, 13 000* g*; 3 min). The filtrate was collected and DNA purified and concentrated in 20 µl using Zymo DNA Clean and Concentrator (Zymo Research, UK) and used as a template for PCR amplification of signature sequences (For Schematic see Fig. S2).

### Amplification of signature sequences

Purified DNA samples (10 µl) from the phage lysate were used as template for amplification of signature sequences using previously published methods. For identification of mycobacteria from the MTB complex, either amplification of IS*6110* by PCR (Eisenach *et al.*, 1990) or of IS*6110* and IS*1081* by RPA was used. For the identification of MAP, IS*900* MAP‐specific PCR method was used (Slana *et al.*, 2009). All PCRs were carried out using Hot Start DNA Polymerase Master Mix Plus (Qiagen, UK).

### Sample collection and ethical considerations

The study protocol was approved by the University of Nottingham, School of Veterinary Medicine and Science ethical review panel prior to sample usage. Blood samples from Single Comparative Cervical Intradermal Tuberculin (SCCIT) reactor cattle were obtained at slaughter. Blood samples were also obtained from live, farmed cattle according to the procedures set out in ‘Exceptional private use of non‐validated tests for TB on cattle in England’ Defra TR496 (Rev. 01/18). Blood samples were obtained from calves experimentally infected with MAP at the Moredun Research Institute as described previously (Watkins *et al.*, 2010). The study design and experimental procedures were assessed and approved by an Ethics Committee and authorized under the Animals (Scientific Procedures) Act 1986.

### Statistical analysis

Statistical analyses including Pearson’s correlations, t‐tests, sensitivity and specificity data and confidence intervals derived from contingency tables were carried out using Prism 7 (GraphPad, San Diego, CA, USA).

## Conflict of interest

The authors declare that the Actiphage^®^ technology described in this paper has been patented (Rees and Swift; PCT/GB2014/052970; European patent No. EP 3052650; US Patent 10,344,339) and that CR and BS are Directors at PBD Biotech Ltd, the company commercializing the Actiphage^®^ technology.

## Supporting information


**Figure S1.** Determination of eclipse phase of D29 infection of M. smegmatis, MAP and M. bovis BCG.
**Figure S2.** Schematic of One Day Method.
**Table S1.** Detection of MTB complex cells using the of the One Day, phage assay and culture of naturally TB infected cattle.
