# Supplementary material for: The development and use of Actiphage® to detect viable mycobacteria from bovine tuberculosis and Johne’s disease‐infected animals
Source: Microb Biotechnol. 2019 Dec 3;13(3):738–46. doi: 10.1111/1751-7915.13518 (PMC7111073; doi:10.1111/1751-7915.13518)
Supplement: Supplementary file 1 — Figure S1. Determination of eclipse phase of D29 infection of M. smegmatis, MAP and M. bovis BCG. Figure S2. Schematic of One Day Method. Table S1. Detection of MTB complex cells using the of the One Day, phage assay and culture of naturally TB infected cattle. [file MBT2-13-738-s001.pdf]

Title

A new method to detect and identify viable mycobacterial pathogens in clinical blood samples within 6 h

Authors

Benjamin M. C. Swift[1#], Nathan Meade[2], Elsa Sandoval Barron[3], Malcolm Bennett[3], Richard J. Sibley[4], Valerie Hughes[5], Karen Stevenson[5], Catherine E. D. Rees[2]

Supplementary materials

Figure S1. Determination of eclipse phase of D29 infection of *M. smegmatis*, MAP and *M. bovis* BCG

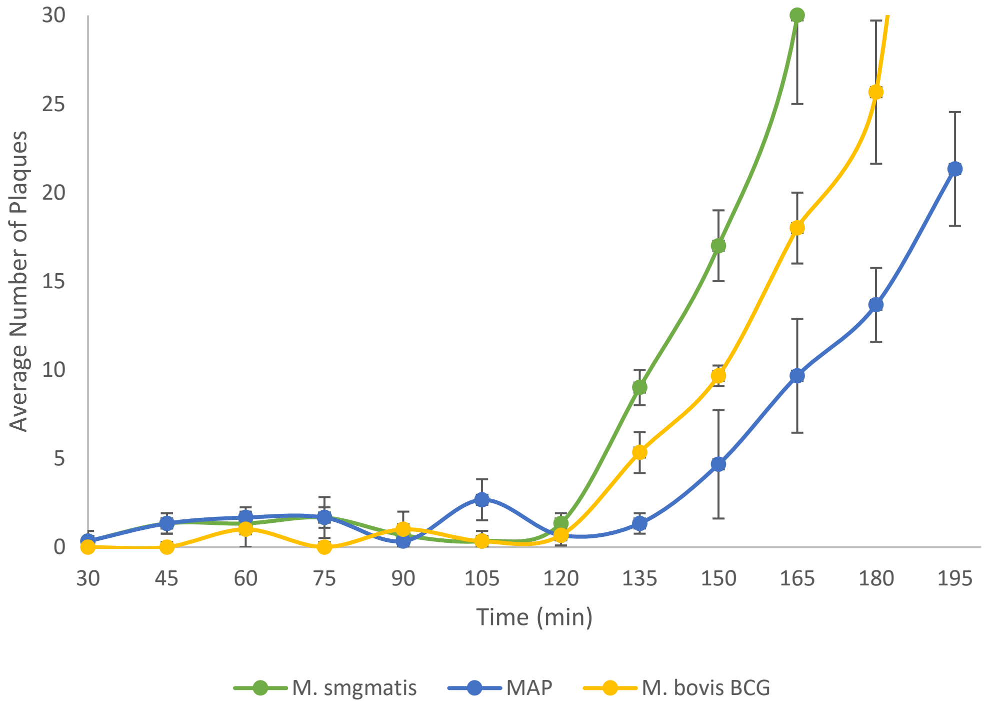

Graph showing the time taken for new D29 bacteriophage virions to be released from *M. smegmatis* (green), MAP (blue) and *M. bovis* BCG (orange). Samples were taken after an initial incubation of 30 min to allow phage adsorption to the host cells. Error bars represent the standard deviations of the means of number of plaques recovered from the phage assay performed in quadruple. The eclipse phase is defined as the time taken for new particles to be released from the cells after infection, thus the period when no phage are detected outside of the host cell.

Figure S2. Schematic of One Day Method

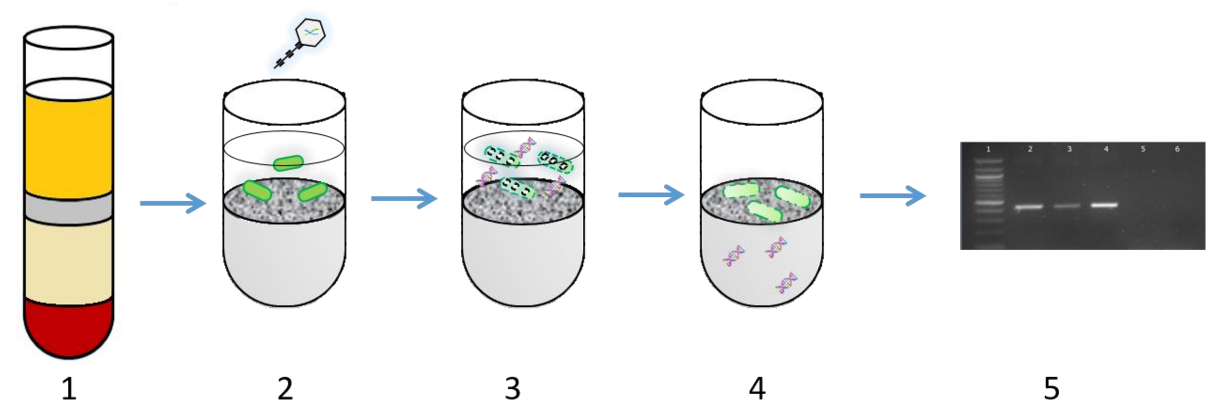

One Day method begins with the preparation of PBMCs from blood sample (1), which is placed into the top half of a filtered tube, and phage are added (2). Sample is incubated at 37 °C for 3 h to allow phage to infect, replicate and lyse their host (3). Sample is centrifuged through filter, separating released mycobacterial DNA from large cell debris (4). DNA is then cleaned and concentrated before amplified by PCR (5).

Table S1 Detection of MTB complex cells using the of the One Day, phage assay and culture of naturally TB infected cattle

Sample Number	No. of Plaques	IS6110 RPA result	One Day Method	PM Result	Culture
**1**	63	+ve	+ve	VL	-ve
**2**	27	+ve	+ve	NVL	-ve
**3**	43	+ve	+ve	VL	-ve
**4**	22	+ve	+ve	VL	-ve
**5**	60	+ve	+ve	VL	-ve
**6**	36	+ve	+ve	VL	-ve
**7**	11	+ve	+ve	VL	-ve
**8**	14	+ve	+ve	VL	-ve
**9**	35	+ve	+ve	VL	-ve
**10**	15	+ve	+ve	NVL	-ve
**11**	14	+ve	+ve	NVL	-ve
**12**	25	+ve	+ve	NVL	-ve
**13**	43	+ve	+ve	NVL	-ve
**14**	26	+ve	+ve	VL	-ve
**15**	3	+ve	+ve	NVL	-ve
**16**	4	+ve	+ve	NVL	-ve
**17**	5	+ve	+ve	NVL	-ve
**18**	20	+ve	+ve	NVL	-ve
**19**	7	+ve	+ve	NVL	-ve
**20**	0	NA	+ve	NVL	-ve
**21**	2	+ve	+ve	NVL	-ve
**22**	0	NA	+ve	NVL	-ve
**23**	16	+ve	+ve	VL	-ve
**24**	0	NA	+ve	VL	-ve
**25**	8	+ve	+ve	VL	-ve
**26**	0	NA	-ve	NVL	-ve
**27**	8	+ve	+ve	NVL	-ve
**28**	7	-ve	+ve	NVL	-ve
**29**	0	NA	+ve	NVL	-ve
**30**	24	+ve	+ve	NVL	-ve
**31**	16	-ve	-ve	NVL	-ve
**32**	0	NA	+ve	NVL	-ve
**33**	0	NA	+ve	NVL	-ve
**34**	0	NA	+ve	VL	-ve
**35**	0	NA	+ve	NVL	-ve
**36**	8	+ve	+ve	NVL	-ve
**37**	32	+ve	+ve	NVL	-ve
**38**	23	+ve	+ve	NVL	-ve
**39**	3	-ve	+ve	NVL	-ve
**40**	1	-ve	+ve	NVL	-ve
**41**	0	NA	+ve	NVL	-ve

No. of plaques – number of plaques observed from the phage-RPA assay

RPA- recombinase polymerase amplification

PM – post-mortem

Culture – denotes the attempt to culture mycobacteria from the clinical blood samples
